# FMEA based prescriptive model for equipment repair guidance

**DOI:** 10.3389/frai.2025.1630907

**Published:** 2025-10-30

**Authors:** Domingos F. Oliveira, Miguel A. Brito, Duarte J. Brandão

**Affiliations:** ^1^Department of Informatics and Computing, Mandume Ya Ndemufayo University, Lubango, Angola; ^2^Centre Algoritmi, Department of Information Systems, University of Minho, Guimarães, Portugal

**Keywords:** multivariate time-series, multi-class classification, FMEA, corrective maintenance, quality assurance and control

## Abstract

**Introduction:**

Accurate prediction of steps required to address machine faults is critical for minimizing downtime and enhancing production efficiency in modern manufacturing. This study utilizes machine failure data and Failure Mode and Effects Analysis to demonstrate how machine learning supports maintenance teams in selecting optimal repair methods.

**Methods:**

The research adopts the Design Science Research paradigm, which emphasizes the creation of artifacts to address practical challenges. For the practical component, quality assurance and control frameworks in data science projects were implemented by integrating two widely used methodologies: CRISP-DM and PDCA, to ensure rigorous quality assurance and control in data science initiatives.

**Results:**

Repair actions serve as the target variables, while the input comprises ten multivariate time-series machine parameters. The prediction task is formulated as a classification problem. Two modeling approaches are evaluated. The first approach merges multiple time series into a single sequence, facilitating the application of Multi-Layer Perceptron, Convolutional Neural Networks, and Fully Convolutional Networks. The second approach preserves the time series as three-dimensional arrays, enabling advanced applications of MLP, CNN, Multi-Head CNN, and FCN models.

**Discussion:**

The models are assessed based on their capacity to predict repair actions, with particular emphasis on the impact of time-series processing and model architecture on classification accuracy. The findings highlight effective strategies for predicting machine repairs and advancing prescriptive maintenance in manufacturing environments.

## 1 Introduction

Intelligent solutions that enhance equipment performance are needed because continuous innovation has accelerated the convergence of technology and operational efficiency in modern business. FMEA is a widely used methodology in several sectors and engineering disciplines to assess, analyze, and reduce risks related to goods, processes, or systems (El-Awady, 2023). The US first used FMEA during World War II in the 1940s.

The FMEA methodology (Jin et al., 2022) has significantly evolved to meet the requirements of diverse industries, such as automotive, electronics, aerospace, and pharmaceuticals. As technological advancement continued, the intricacy of products and processes increased, and most FMEAs significantly contributed to the improvement of product quality, safety, and reliability.

**Figure 1:**
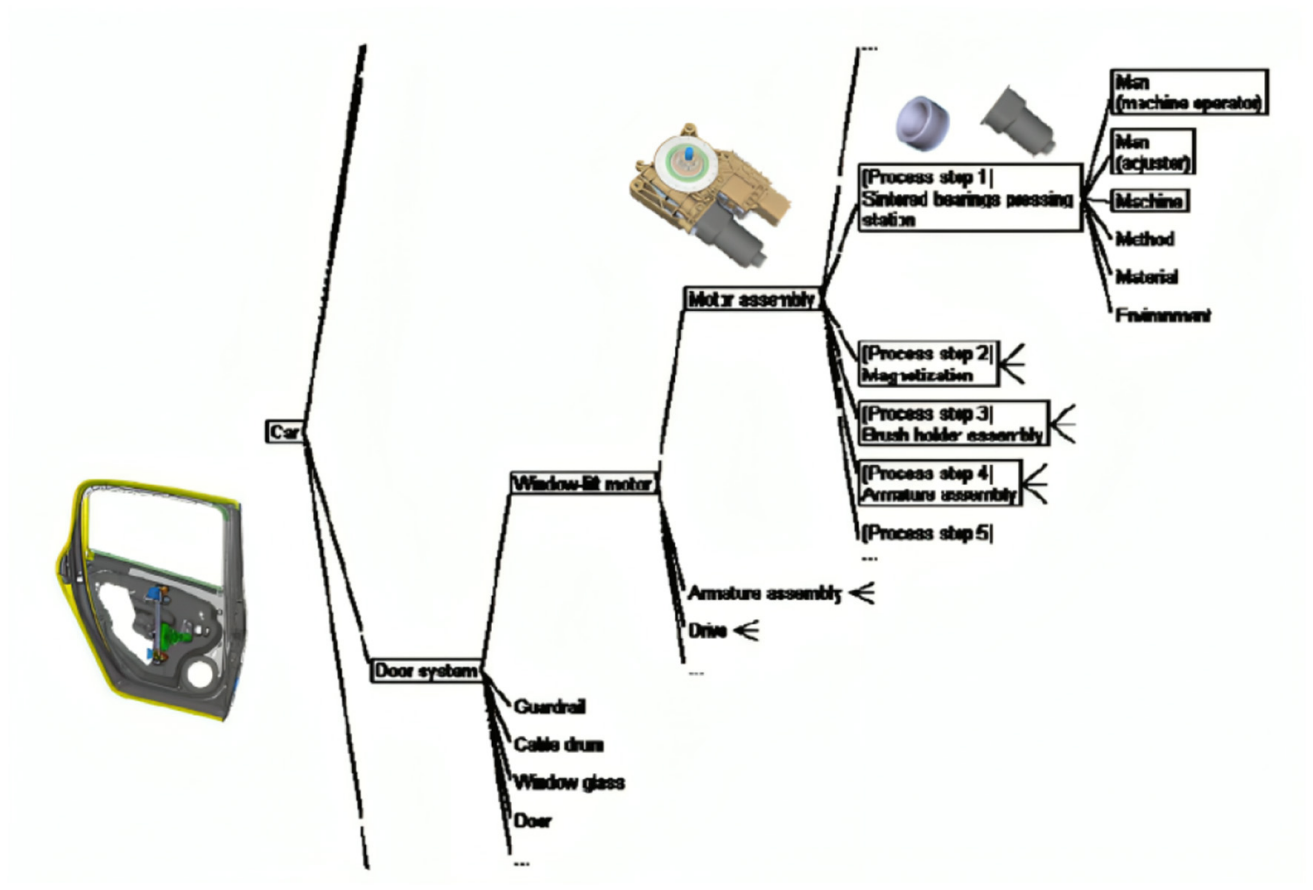
Process FMEA example of a window lift motor.

Bosch Car Multimedia relies on FMEA to identify, assess, and mitigate failure risks to ensure product quality and production efficiency. Two FMEA types: product and process. This final article covers FMEA terminology for systems, interfaces, designs, production, assembly, logistics, and machinery. Product FMEAs analyze a product's quality life cycle, components, and interactions. Process FMEA, shown in Figure 1, assesses quality-related systems and processes from item receipt to consumer delivery.

The picture delineates the assembly process of the window lift motor, highlighting probable failure modes and contributing factors at various stages. This exemplifies a process FMEA methodology, wherein each stage of the assembly is scrutinized for risks, utilizing the framework to pinpoint probable failure causes related to human, machine, technique, material, and environmental issues.

The project will focus on process FMEA analysis of manufacturing line equipment maintenance requests. Equipment failure notices trigger a repair order that the maintenance team addresses. Failure cause, effect, action, and other parts of the FMEA analysis will follow.

### 1.1 Motivation

Market competitiveness has led to a pressing need to optimize maintenance processes and increase operational efficiency in the industry. In a scenario where Bosch Car Multimedia plays a crucial role in the manufacture of high-quality automotive equipment, the occurrence of repair machine failures can have significant implications for productivity and product quality.

The desire to anticipate and respond proactively to faults in the industrial machinery deployed on production lines is key to minimizing unplanned downtime. The implementation of a predictive system based on FMEA offers the opportunity to go beyond the conventional reactive approach, allowing Bosch to adopt a prescriptive stance in the maintenance of its machines.

In exploring this topic, the aim is not only to test different model's for predicting corrective actions, but also to understand the complexities inherent in the variety and imbalance in the classes of corrective actions. Overcoming these challenges will contribute to the advancement of research in artificial intelligence applied to industrial maintenance.

The motivation lies in the vision of creating a more efficient production environment where repair machines not only identify faults, but with the help of artificial intelligence suggesting precise corrective actions in real time. This approach will not only boost operational efficiency by reducing costs associated with downtime but will also strengthen Bosch's reputation as a leader in innovation and quality in the automotive industry.

### 1.2 Objectives

This research develops prediction algorithms to help industrial maintenance teams fix production equipment. The main goal is to combine temporal sensory data from equipment before failures with historical FMEA records describing past equipment failures, their root causes, and corrective actions. This integration will reveal the impact of organized (FMEA reports) and unstructured (sensor time series) historical data.

The project also investigates multi-class classification techniques applied to multivariate time-series data, finds patterns and correlations that predict future failures, and uses machine learning algorithms that can handle complex industrial environments where many interdependent variables affect equipment conditions. Another goal is to compare machine-learning and time-series data preparation methods. These methods will be tested for their ability to predict remedial procedures based on prior failures.

The focus will be on methods that handle the complex relationships between sensory data, equipment failure modes, and maintenance procedures. The models' ability to recommend remedial actions for each failure scenario will be emphasized. The comparison study determines the best forecast accuracy and real-world applicability of maintenance team suggestions. If the models work, a real-time data pipeline will be created for predictive maintenance in active production.

### 1.3 Methodologies

The article does not provide a very clear description of the research and discussion process; please provide a detailed description. The research was developed using Design Science Research, a research paradigm focused on creating artifacts (models, methods, tools) to solve practical problems, while contributing to the advancement of scientific knowledge (vom Brocke et al., 2020).

**Figure 2:**
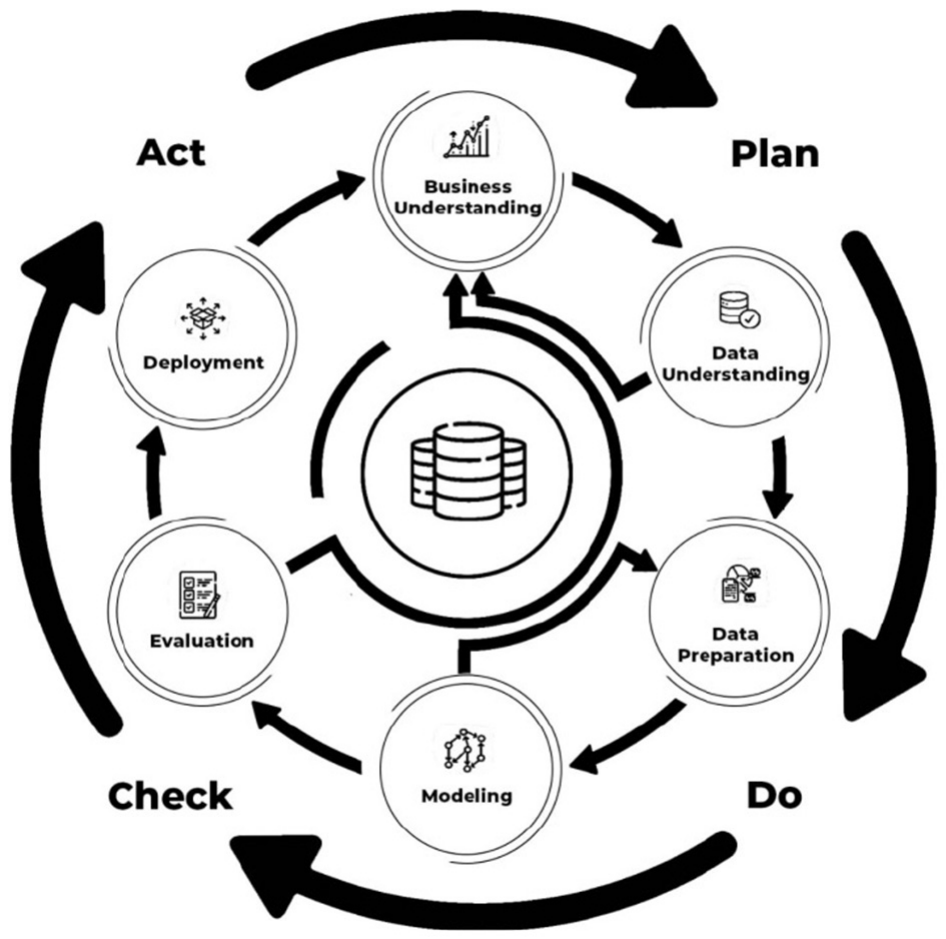
Framework for quality assurance and control in DS projects adapted from Oliveira and Brito (2023).

For the practical part, quality assurance and control frameworks in data science projects were used, which sought to combine two widely used tools, namely CRISP-DM and PDCA, to perform quality assurance and control in DS projects. Since the project falls within the areas of data science, it aims to provide measures to predict appropriate repair activities for industrial equipment during corrective maintenance (Oliveira and Brito, 2023). This method (Figure 2) provides a systematic framework for the development of artifacts and the optimisation of industrial repair.

### 1.4 Article structure

This document presents a structured methodology for forecasting corrective maintenance operations using machine learning techniques applied to multivariate time series data. The introduction outlines the research context, objectives, and scope. It focuses on predicting optimal remedial actions for production line failures. The subsequent sections detail the materials and methods. They include a literature-based overview of Bosch's maintenance processes and an emphasis on key topics. Remedial measures are discussed within the Framework for Quality Assurance and Quality Control in Deep Learning Systems (Oliveira and Brito, 2023). This framework is pertinent to data science projects.

The document describes several modeling scenarios that use similar machine learning models. These models are primarily differentiated by their architectures and how they structure time series data. The fourth section presents the main findings tied to the research objectives stated in the introduction. It also evaluates the degree to which these objectives were achieved. Additionally, it assesses the performance of predictive models developed with machine learning techniques on multivariate time series data. These results show the potential of such models to help maintenance teams. They can recommend effective repair strategies for manufacturing line problems.

The fourth section presents results through a comparison of two scenarios and proposes directions for future research. The conclusions and recommendations provide a comparative analysis of data from the two modeling methodologies, address broader implications, and discuss the potential for integration with Bosch maintenance systems. The study identifies limitations including data availability, variability in model performance, and computational challenges, thereby offering a realistic assessment of constraints.

## 2 Materials and methods

In any machine learning endeavor, particularly in classification tasks, assessing model performance is crucial to guarantee its trustworthiness and efficacy. The selection of evaluation metrics is contingent upon the problem's characteristics, class distribution, and the precise objectives of the prediction model. This study analyzes multi-class categorization of corrective activities utilizing past failure and sensor data, employing various key criteria to assess the model's efficacy in forecasting appropriate repair operations.

### 2.1 Label reduction using text clustering

In text clustering, Subakti et al. (2022) study the effectiveness of BERT in text representation for unsupervised learning tasks, focusing on text clustering. The essay describes BERT text extraction, including tokenization, padding, and encoding. Comparative assessments of text data representations from AG News, Yahoo! Answers, and R2 highlight metrics. The research attributes BERT's success to its ability to align related texts.

Deep Feature-Based Text Clustering (DFTC) is a novel text clustering structure proposed by Guan et al. (2022). Examining whether deep text encoders are appropriate for text clustering, contrasting the suggested model with traditional text clustering models, and presenting a Text Clustering Results Explanation (TCRE) model to assess the clustering outcomes qualitatively are the main objectives. Traditional text clustering techniques, including Gibbs Sampling Dirichlet Mixture Model (GSDMM), Latent Dirichlet Allocation (LDA), tf-idf-based K-means, and a cutting-edge pre-trained language model, BERT, are compared.

Overall, the DFTC framework, with its deep text encoder and TCRE model, is presented as a robust and effective approach for text clustering, outperforming both traditional and other deep learning-based models in the experiments conducted.

### 2.2 Feature reduction

The paper Subasi et al. (2024) carefully analyses and identifies feature reduction methods that have low costs/overheads in terms of time and memory. The identified reduction methods are also evaluated in terms of their impact on the accuracy, precision, time and memory costs of traditional classification algorithms. Specifically, we focus on the less resource-intensive feature reduction methods that are available in the Scikit-Learn library. In the evaluation, it was found that in quadratic-scale feature reduction, the classification algorithms achieve the best compromise between competing performance metrics. The results show that overall training times are reduced by 61%, model sizes are reduced by 6 × and accuracy scores increase by 25% compared to baselines, on average, with quadratic scale reduction.

In label reduction, Siblini et al. (2021) analyzes over 50 publications to study dimensionality reduction strategies in multilabel classification. There are three primary methods: reducing the feature space and predicting the label matrix, diminishing the label space, forecasting the reduced label matrix, and simultaneously diminishing both. Conditional Principal Label Space Transformation (CLPST) was one of the first feature-based label reduction methods.

### 2.3 Technics for multi-class classification

The study Rácz et al. (2021) the effects of dataset size and training/test split ratios on various machine learning classification models were meticulously examined through three distinct case studies. The research involved repeated modeling with different versions of starting datasets, varied numbers of samples (NS), and train/test split ratios (SR), employing five iterations for each combination of these parameters. The approach integrated detailed analyses of variance (ANOVA) and multicriteria evaluations to elucidate the influence of these factors on the performance of multiclass classification models.

The findings led to a recommendation for the use of an 80%/20% training/test split ratio, particularly for larger datasets, ensuring an ample supply of training samples for multiclass classification. This comprehensive analysis sheds light on the nuanced interactions between dataset characteristics and machine learning model performance, providing valuable insights for practitioners navigating the intricacies of classification tasks.

### 2.4 Multivariate time-series with multiclass classification

Taco et al. (2024) proposes a unique method for categorizing failure modes by extracting characteristics from multivariate time series data. Deep learning's algorithmic complexity, hardware requirements, and extensive training times inspired this alternate method. The CNN model outperforms deep learning techniques with an accuracy of 82% in the frequency domain. In the frequency domain, the LSTM, BiLSTM, and ConvLSTM models achieve peak accuracies of 76%, 76%, and 81%.

In van den Hoogen et al. (2021), deep learning methods enhance defect identification and condition monitoring, notably for rolling bearing parts. The study classifies spinning machinery multivariate data using one-dimensional CNNs. The models are tested across multiple datasets with various contexts and training data to demonstrate their generalizability.

The Time Series Attentional Prototype Network, introduced in Zhang et al. (2020), is a novel multivariate time series classification model that extracts low-dimensional features without domain knowledge and addresses the issue of limited labeled data. By rebuilding time series dimensions into groups using convolutional layers, the authors suggest a random group permutation method to discover latent features efficiently.

The Baldán and Benítez (2021) study examines traditional models with two objectives: producing interpretable data and optimal classification outcomes, notwithstanding potential interpretability issues. The technique shows each MTS variable's attributes. Transforming the original dataset by arranging its variables in a row creates a new dataset that combines the properties of all MTS variables in one instance.

The work presented by Fu and Liu (2022) suggests a data-driven method for predicting the low-voltage contactor's remaining useful life (RUL). The three-phase alternating voltage and current are used to record the electrical equipment's useful life and track how many times it has been used. Then, using the time domain, frequency domain, and wavelet methods, the characteristics that are relevant to the failure are extracted. A CNN-LSTM network is then designed and used to train an RUL prediction model for the electrical equipment based on the extracted features. The results show that the suggested method performs better than the most popular deep learning algorithms in terms of MAE and RMSE.

The problem with fault detection applications is that data from a single sensor may not be sufficient in terms of performance to detect abnormalities in equipment, as discussed in the work of Kullu and Cinar (2022), predictive maintenance. This research suggests a deep learning approach based on multimodal sensor fusion to address this issue by combining data from various signal domains and sensors to identify equipment failures. The short-term Fourier transform (STFT) is used to convert raw vibration and current sensor data into time-frequency pictures. The deep learning model, intended to identify flaws, was then fed the time-frequency images and raw time series data. The findings demonstrated the potential benefits of employing the suggested approach for multimodal sensor data fusion in the identification of equipment malfunctions.

To reduce the error of equipment operation trend prediction, Wang et al. (2023) proposes a method for equipment operation trend prediction based on a combination of signal decomposition and an Informer prediction model. Aiming at the problem of high noise in vibration signals, which makes it difficult to obtain intrinsic characteristics when directly using raw data for prediction, the original signal is decomposed once using the variational mode decomposition (VMD) algorithm optimized by the improved sparrow search algorithm (ISSA) to obtain the intrinsic mode function (IMF) for different frequencies and calculate the fuzzy entropy. The improved adaptive white noise complete set empirical mode decomposition (ICEEMDAN) is used to decompose the components with the largest fuzzy entropy to obtain a series of intrinsic mode components, fully combining the advantages of the Informer model in processing long time series and predicting equipment operation trend data. The experimental results indicate that the proposed method can effectively improve the accuracy of equipment operation trend prediction compared to other models.

Using three data sets about the operation of (1) an industrial wrapping machine operating in discrete sessions, (2) an industrial blood refrigerator operating continuously, and (3) a nitrogen generator operating continuously, this study Pinciroli Vago et al. (2024) assesses the effects of reading window and prediction window size on the performance of models trained to forecast failures. Multivariate telemetry time series are used to compare six algorithms: Transformers, LSTM, ConvLSTM, Random Forest, Support Vector Machine, and Logistic Regression. The findings show that the dimension of the prediction windows is important in the scenarios under consideration. They also demonstrate how well DL approaches classify data with a variety of time-dependent patterns before a failure, while ML approaches classify similar and repeating patterns.

### 2.5 Metrics

In machine learning, especially in classification tasks, model performance must be evaluated to ensure reliability and effectiveness. Evaluation metrics depend on the characteristics of the problem, the class distribution, and the model's objectives. This article uses previous failure and sensor data to categorize corrective efforts into multiclass categories and evaluates the model's ability to predict repair operations using crucial metrics. Thus, for this work, only accuracy will be explored, leaving other metrics for future work.

- $Accuracy=\frac{TP+TN}{TP+TN+FP+FN}$;
- $Precision=\frac{TP}{TP+FP}$;
- $Recall=\frac{TP}{TP+FN}$;
- $F1=\frac{2*Precision*Recall}{Precision+Recall}$.

Where:

- TP (True Positives): The number of positive instances correctly classified by the model.
- TN (True Negatives): The number of negative instances correctly classified by the model.
- FP (False Positives): The number of negative instances incorrectly classified as positive by the model.
- FN (False Negatives): The number of positive instances incorrectly classified as negative by the model.

### 2.6 Maintenance process flow

**Figure 3:**
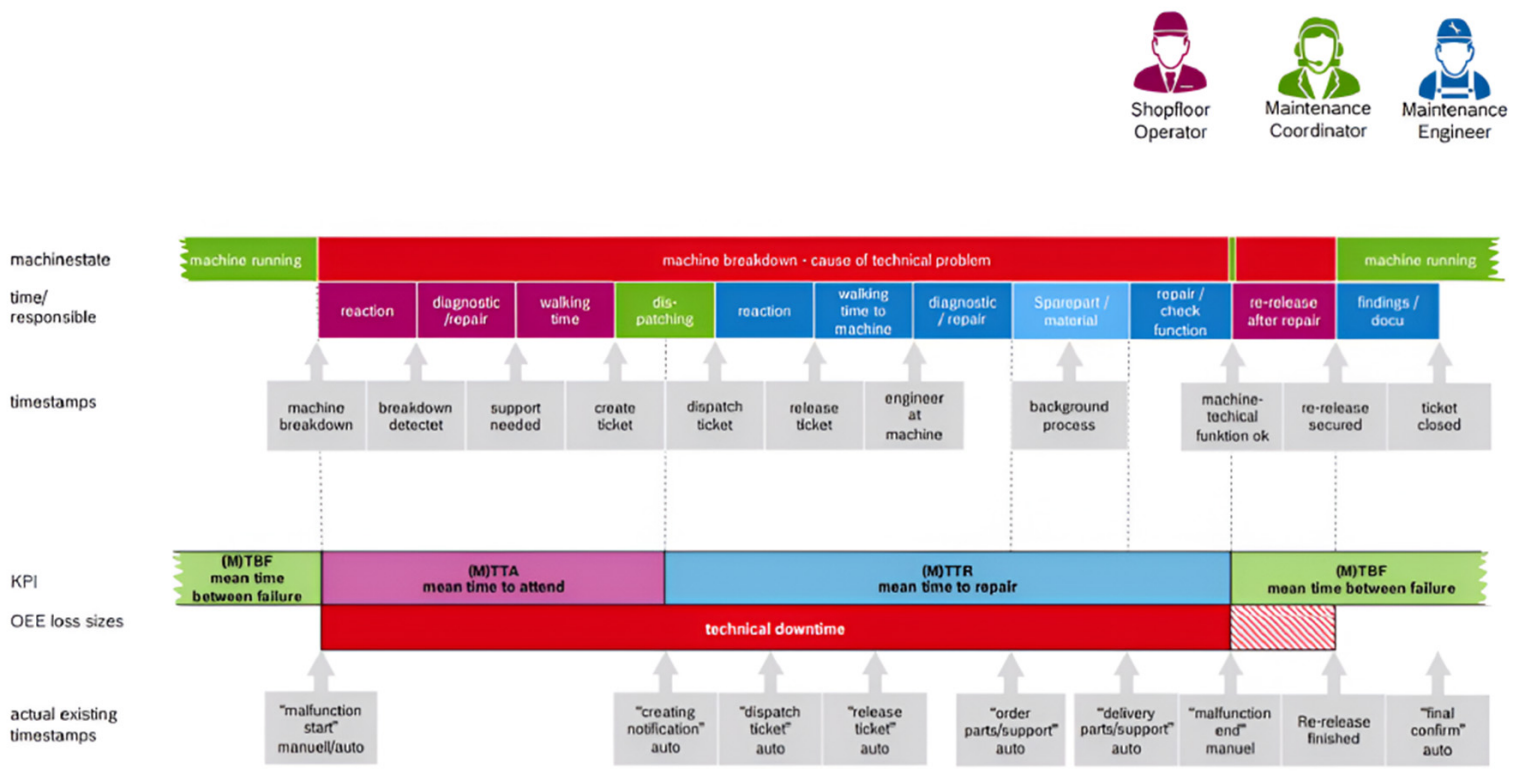
Corrective maintenance MTBF, MTTA, and MTTR KPI's workflow.

The company's corrective maintenance process includes machinery, shop floor workers, maintenance teams, and IT systems, each having subprocesses and decision points. The maintenance team evaluates corrective fault repair performance using three key performance indicators (KPIs): Mean Time Between Failures (MTBF), Mean Time to Acknowledge (MTTA), and Mean Time to Repair (MTTR) (see Figure 3). This effort aims to improve MTTR by reducing equipment repair.

The workflow provides an overview of key performance indicators (KPIs), such as MTBF (average time between breakdowns), MTTA (average response time to breakdown), and MTTR (average time needed for repairs). The graph also shows OEE loss sizes, which record the damages caused by equipment failure and offer dates for different phases of the maintenance procedure.

### 2.7 Maintenance IT system

BCore IT System was built for the project. The web-based system manages real-time manufacturing, maintenance, and other tasks. BCore manages maintenance order creation, status updates, and selections. This system keeps complete records of all maintenance orders. BCore calculates MTBF, MTTA, and MTTR from maintenance order status transactions.

### 2.8 Corrective maintenance: bridging the gap

**Figure 4:**
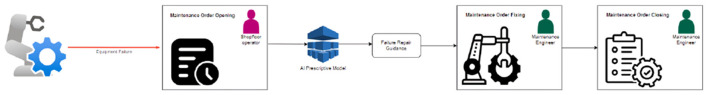
AI prescriptive model application in corrective maintenance process.

This subsection explains the corrective maintenance project framework in Figure 4. The primary goal is to use artificial intelligence to identify the best Failure Mode field for the failure/effect to help the maintenance team fix industrial equipment integrated into the manufacturing line. Maintenance teams can rapidly identify the cause of the fault and determine the proper fix by evaluating the failure mode column.

Implementing AI significantly benefits the maintenance process by streamlining troubleshooting procedures and minimizing downtime caused by equipment malfunctions. With AI assistance, maintenance teams can swiftly identify and address issues, leading to expedited resolutions and reduced operational disruptions. The strategy entails leveraging historical data from corrective maintenance conducted on organized production lines to empower the model in FMEA field outcomes.

We will include production equipment metrics in the model in addition to past corrective maintenance order data. To fully cover this topic, a multivariate time-series multi-class classification model is needed to analyze ten sensor-monitored parameters for the screwdriving system equipment. The Failure Mode column has numerous classes, and this model will categorize it using time-series parameters.

### 2.9 Development

This section describes creating a machine learning model using data science design, quality assurance, and control. The approach improves design and allows maintenance teams to predict production line faults. Time series data on machine attributes is used to predict defect repair actions. The development technique comprises problem identification, exploratory data analysis, data preparation, and modeling two predictive scenarios.

#### 2.9.1 Plan

Robotics has improved production line efficiency and precision in modern manufacturing. These gains come with difficulties, especially in fault detection and correction. Forecasting and fixing equipment defects is crucial to smooth operations and productivity for the company's production lines, where robotic and machine equipment is critical.

##### 2.9.1.1 The problem and its challenges

This work delves into the utilization of time series data gathered from organizations' sensors installed in robotic equipment to classify corrective actions for identified faults. Yet, despite this approach's promise, numerous challenges must be overcome to effectively predict and implement corrective actions in real-world manufacturing environments.

Identifying and diagnosing robotic equipment failures is difficult. Interconnected robotic systems with sensors capture many data types on production lines. To find the core cause of defects in these different data streams, sophisticated algorithms must identify regular operating changes from prospective breakdowns.

##### 2.9.1.2 Phenomenon analysis

Robotic equipment operates in unpredictable circumstances, affecting product specifications, manufacturing volumes, ambient conditions, and maintenance schedules. It is crucial to create robust models that can handle variations and accurately predict issues. Production line sensor data may be noisy, partial, or irrelevant, requiring pre-processing and feature engineering. Predictive models require turning sensor data into functional attributes.

AI models struggle with multiple labels and multi-output scenarios. Multiple labels and decision boundaries may increase computing and overfitting. The output space becomes more dimensional in multi-output contexts, complicating learning. Managing output dependencies and correlations gets increasingly complicated, necessitating advanced designs and training methods to capture the linkages.

Forecasting and real-time adaptation complicate things. Corrective activities must be accurately predicted to minimize downtime in continuous manufacturing environments with fixed production timetables. Real-time forecasting requires efficient algorithms and predictive models in the production line architecture. If the above issues are addressed, predictive maintenance solutions can forecast production line robotic equipment malfunction repairs.

#### 2.9.2 Execute

After defining the project's problems, we move on to the project's implementation section. As mentioned in the previous section, the data relating to industrial equipment parameter measurements and the respective failure analyses are stored in a MySQL database using the BCore software developed within the company.

##### 2.9.2.1 Exploratory data analysis

In the MySQL database, two tables record the necessary information. One of these tables is view_maintenanceorders, which records all the maintenance carried out in production (corrective or preventive), and the other is view_measurements, which records some of the equipment parameters on the production lines. Data has been stored in the maintenance since 2020, and the categorization of data related to FMEA only began on September 18, 2023, before corrective maintenance was free text.

The maintenance crew categorized all equipment failures. FMEA fields define corrective issues by failure effect, mode, cause, and function. This table covers all corrective incidents, but will focus on 'Robot Universal' equipment because it has the most precise FMEA data linked with maintenance activities. From “2023-09-18 06:00:00” to “2024-02-29 23:59:59”, data in the view_maintenanceorders and view_measurements tables was filtered for “Robot Universal” equipment subtype.

Given the range of categories in the failure effect column, the category “Falha de Aparafusamento” is used to evaluate the categorization capabilities of the other fields (failure mode, FMEA cause, FMEA action) while completing a correction order for the failure/effect in universal robots. However, the few cross-links show that fmeacause and fmeaaction are not diverse. Once a repair order's failure mode is recognized, the maintenance operator can easily determine its FMEA cause and response.

The maintenance team corroborated this result, emphasizing that the crucial part is identifying the failure mode associated with the remedial event. In the table view_measurements, only robots documented in view_maintenanceorders with data records for the same timeframe between “2023-09-18 06:00:00” and “2024-02-29 23:59:59” were chosen for merging. Before merging with the maintenance orders database, the data needs preprocessing to guarantee temporal synchronization of the recorded parameters.

**Figure 5:**
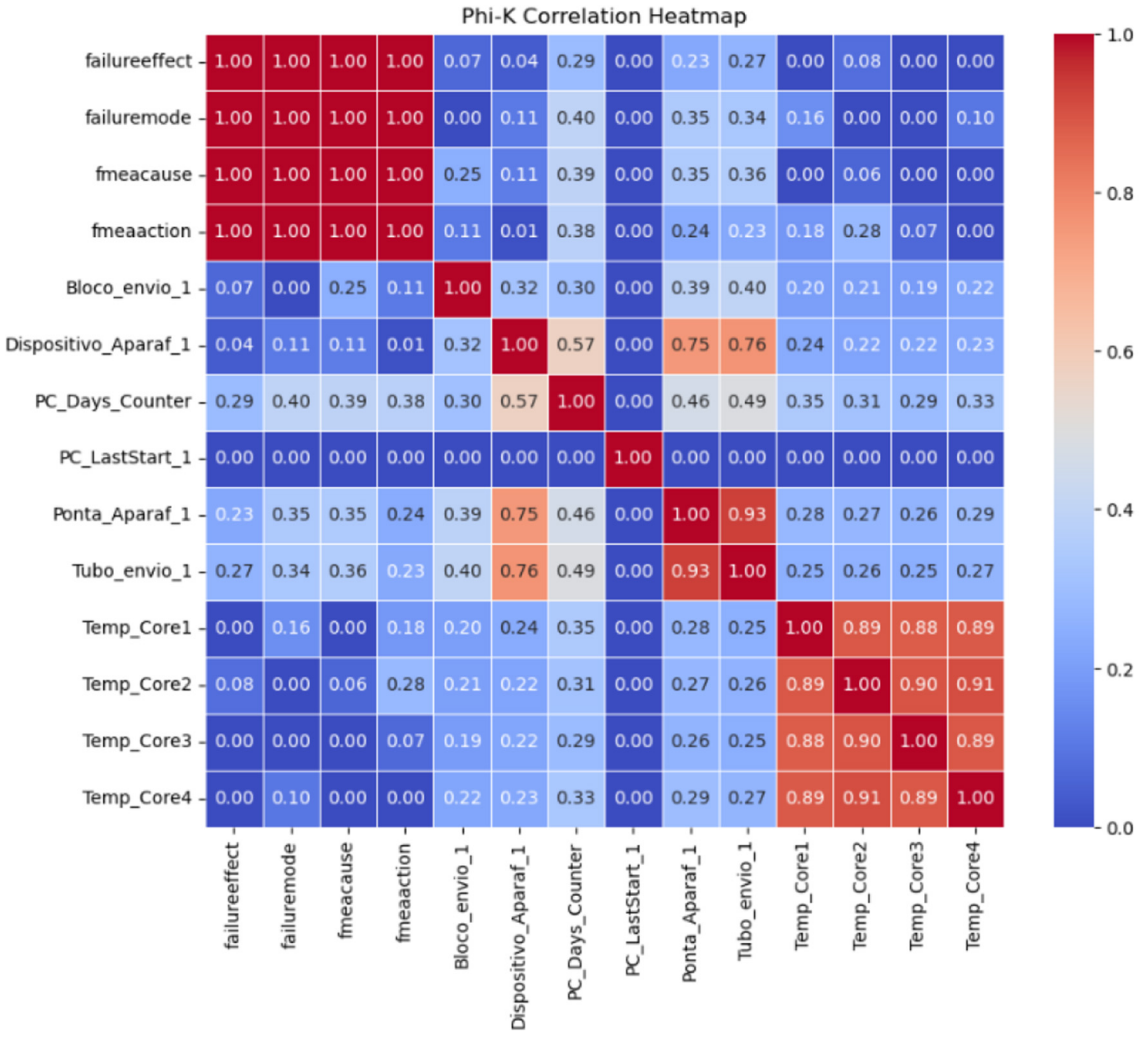
Phi_k correlation result between parameters and FMEA columns.

This table includes 10 parameters relating to the universal robot's computer-sensed measurements. A correlation graph, Figure 5 was created using the Phi_k coefficient to analyze the correlation between categorical and numerical variables. This method is particularly effective for assessing relationships in mixed data types, providing a robust measure of association.

The Phi_k coefficient, Adriani and Palupi (2021) is designed to handle the complexities of mixed data types, making it a versatile tool for modern data analysis. Phi_k operates by first transforming categorical variables into numerical counterparts through an optimal binning process. This process ensures that the categorical data is represented to maximize information retention and the original data's statistical integrity.

##### 2.9.2.2 Data preparation

This section details the data preparation steps undertaken before the data modeling phase. It includes data cleaning, transformation, and normalization processes, handling missing values, and encoding categorical variables. As each parameter is not recorded simultaneously, it was necessary to group the data into 15-minute intervals. The next step involved joining the two tables based on the maintenance dates and when the curve values for each parameter were recorded.

Standardizing failuremode column entries improved classification model performance and analysis clarity. The original dataset included class labels for the same failure mode but different labels. Redundancy adds complexity and noise, which may affect model performance. Therefore, these classes were renamed and merged into more consistent groups. This preprocessing procedure simplified the dataset and enhanced the model's failure mode learning and prediction.

Besides these modifications, the equipment's 10 parameters' time series data includes holidays and production slowdowns. The study and results can be significantly affected by these data gaps. To maintain a continuous dataset, interpolation techniques (Oh et al., 2020) are used to fill in missing values. Interpolating the data preserves the time series and improves analysis.

The duration of corrective maintenance for equipment varies, leading to time series with different lengths. To standardize these time series and ensure they all have the same number of time steps, we removed the last x time steps before corrective maintenance. However, it was essential to filter out only those time series that contained at least x time steps before the maintenance event. This approach ensures that all included time series are of uniform length, thereby enabling consistent and reliable analysis across the dataset.

Time series data with 100, 500, 1000, 1500, and 2000 time steps were tested to analyze various scenarios. The time series were structured in two ways. The first method concatenated a 10-parameter time series horizontally into one extended time series. The second method used three-dimensional arrays of time series. This 3D array preserves temporal dependencies and interactions between variables across samples for sophisticated modeling approaches like deep learning, enabling more complicated and detailed analysis.

##### 2.9.2.3 Data modeling

This phase of our study aims to construct robust and precise models for multivariate time-series multiclass classification. Our methodology will utilize three separate modeling techniques: Multi-Layer Perceptron, Convolutional Neural Networks, and Fully Convolutional Networks. To thoroughly assess the performance and efficacy of these models, we will employ time-series datasets of varied lengths: 100, 500, 1000, 1500, and 2000 time points over the previously outlined scenarios.

This data modeling phase aims to create and evaluate the performance of MLP (Arablouei et al., 2023), CNN (Khushi et al., 2024), and FCN (Agarwal et al., 2023) models on time-series datasets of varying lengths and contexts. Through a comprehensive evaluation of these models, we want to discern the strengths and weaknesses of each method in managing multivariate time-series data for multiclass classification problems.

Before implementing the models, it is essential to understand how the class distribution of the time series varies with size. As the size increases, the number of classes tends to decrease. This happens because many samples might not meet the size requirement. Specifically, data may not be available for a given size if a corrective failure occurs.

## 3 Results

This section examines the results obtained through the experiments from the methodologies elucidated in the previous sections and is divided into two scenarios.

### 3.1 Modeling—Scenario 1

As described in the data preparation section, the preliminary scenario entails classifying the failure mode column using 10 distinct time series parameters. The MLP was tested first, with results from five time series sizes filtered to 500 steps. Thus, the final time series with all 10 parameters will have 5,000 steps (500 * 10). Basic MLP and Keras auto-tuner MLP performance was compared.

**Figure 6:**
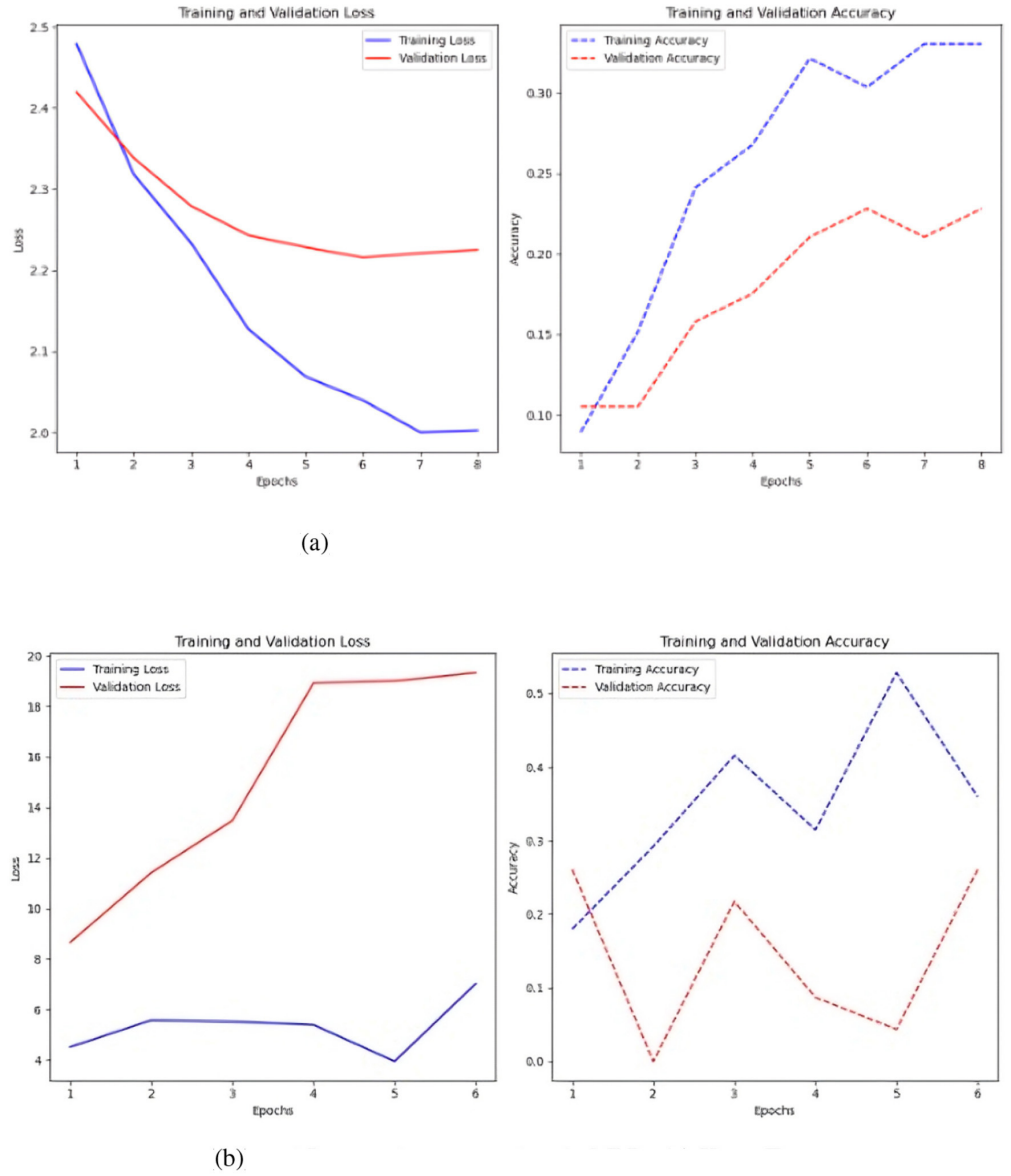
Represents the value of the loss over the seasons and the value of the accuracy. **(a)** Sc 1 loss and accuracy values in MLP. **(b)** Sc 1 loss an accuracy values in MLP with Keras-Tuner.

The optimal outcome with a basic MLP was attained using 1,500 time steps per time, resulting in a test loss of 6.59 and an accuracy of 0.25. The Keras auto-tuner yielded optimal results with 500 time steps, achieving a loss of 2.23 and an accuracy of 0.32, as depicted in Figure 6.

Figure 6b shows that the loss in the simple MLP is stable and low over epochs, indicating effective learning of the training data. However, this may indicate issues with the training data itself. While the accuracy fluctuates, the training accuracy typically increases, reaching 0.5, indicating superior training data identification. Validation accuracy is best around 0.3, changing throughout epochs.

The MLP with Keras auto-tuner (Figure 6a) significantly improved training and validation loss, demonstrating effective performance and no overfitting. The minor difference between training and validation loss reflects good learning in the model. Training accuracy of 0.35 and validation accuracy near 0.25 indicate that the model's performance increases with more training epochs.

**Figure 7:**
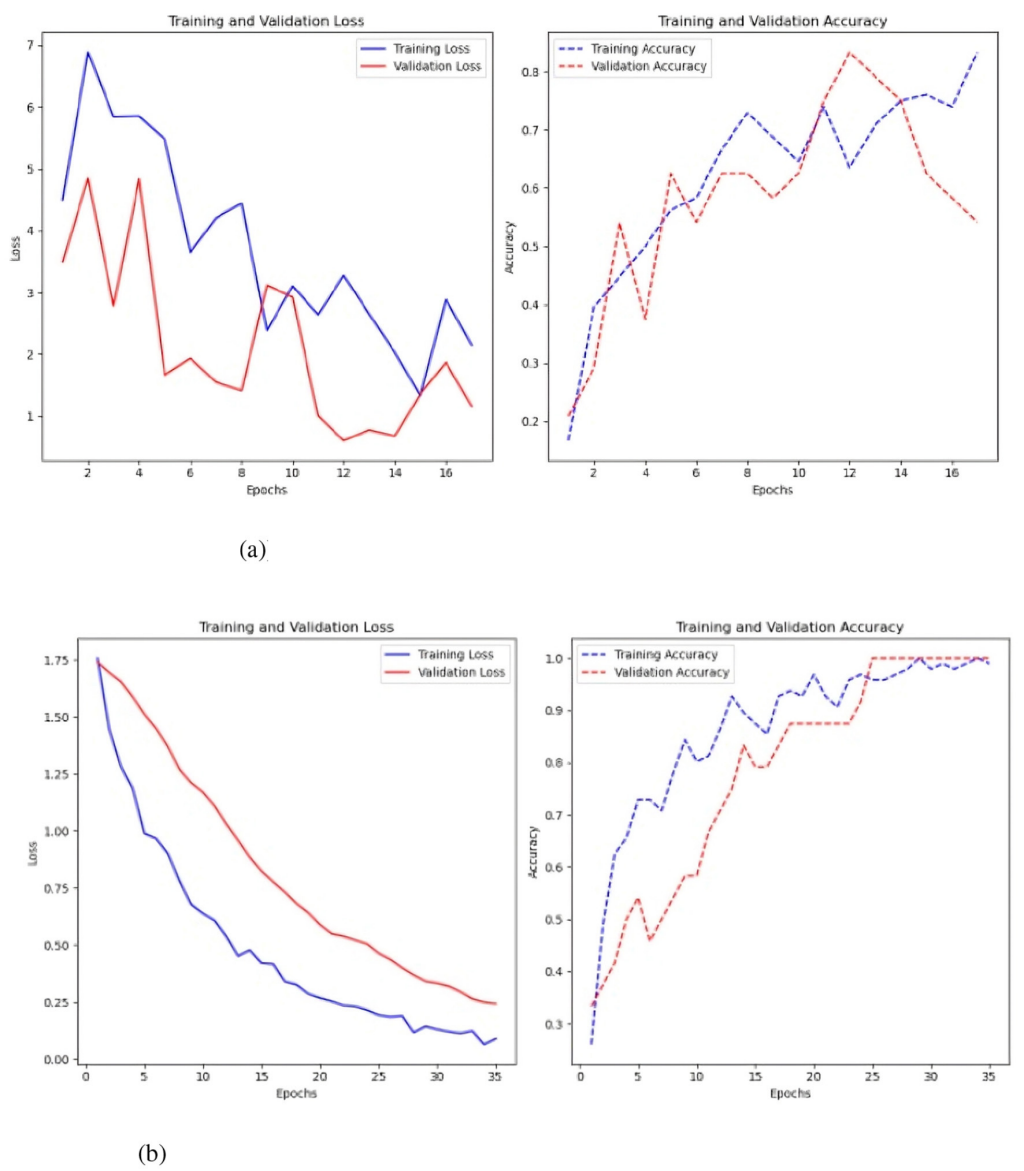
Results of class balancing using SMOTE. **(a)** Sc 1 loss and accuracy values in MLP with SMOTE. **(b)** Sc 1 loss an accuracy values in MLP with Keras auto-tuner and SMOTE.

A test was conducted to balance the classes utilizing SMOTE (Figure 7), and the findings indicate that, although an overall decline in performance, a significant enhancement was observed when the time series were constrained to 2000 time steps. The test accuracy was 0.25, and the loss was 10.251 with a fundamental MLP. The test accuracy was 0.20, while the test loss was 2.54 when employing the Keras auto-tuner.

**Figure 8:**
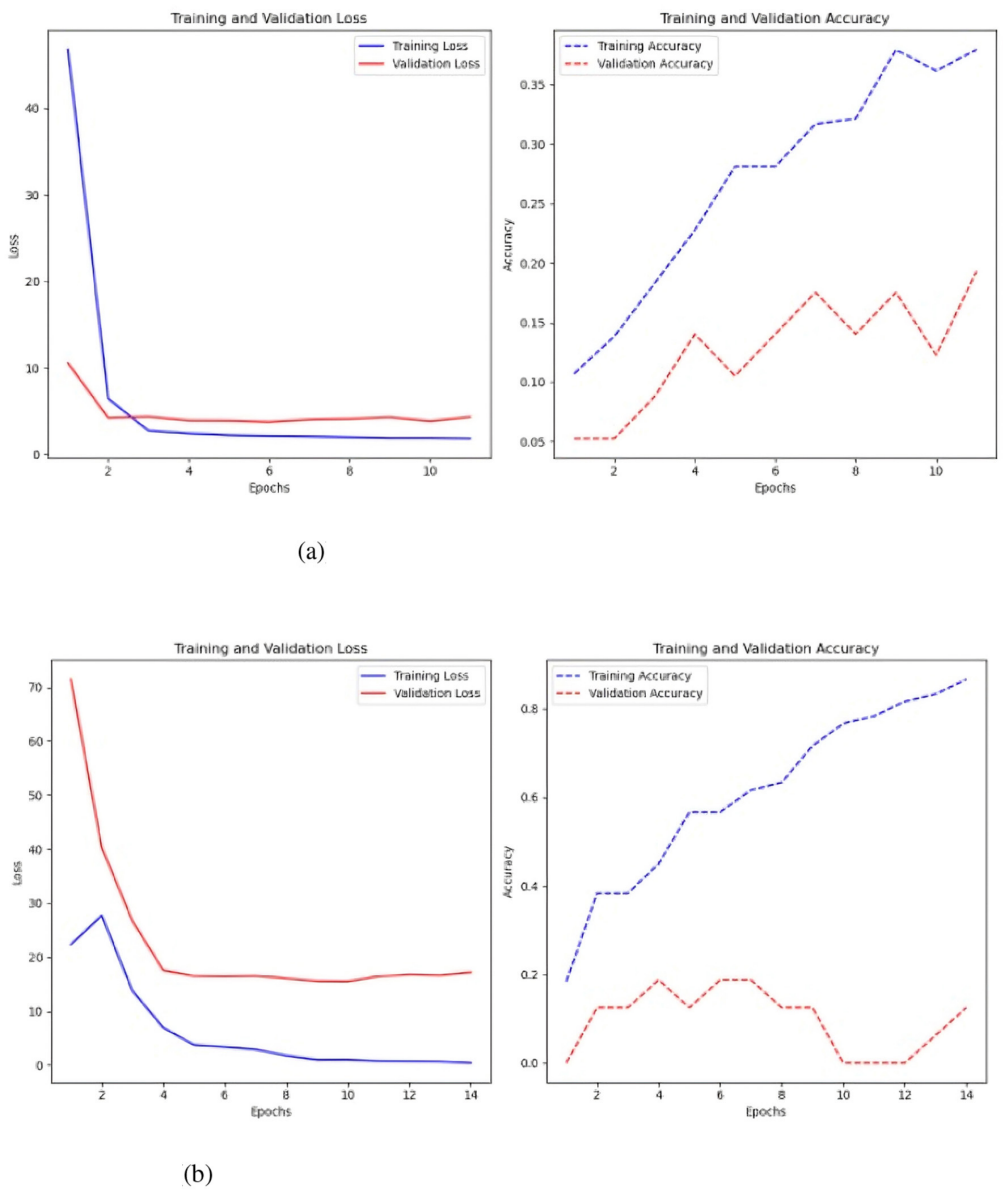
Results of the CNN model. **(a)** Sc 1 loss an accuracy values in CNN. **(b)** Sc 1 loss an accuracy values in CNN with Keras auto-tuner.

The second model assessed was a CNN. This model was evaluated with and without the Keras auto-tuner used to improve hyperparameters (Figure 8). 200-time steps per parameter produced the best results with a simple CNN architecture (Figure 8b), with an accuracy of 0.30 and a loss of 4.13. The model processed 500 data per parameter when the CNN parameter optimization Keras auto-tuner was used. This led to a much lower loss of 2.29 and a somewhat better accuracy of 0.338.

The CNN's training loss decreases as the model progresses, indicating effective convergence. The validation loss stabilizes and marginally rises after epoch 6, signifying overfitting. The validation loss fluctuates and stabilizes at about 25.0, whereas the training loss attains a minimum of approximately 5.0. The training accuracy steadily increases, attaining around 80% by the 14th epoch. The validation accuracy exhibits variability, initially reaching a peak of about 40%, thereafter declining and stabilizing at 20%.

The CNN with Keras auto-tuner achieved better results (see Figure 8a), with training loss reducing and stabilizing at zero, indicating effective learning. The validation loss rapidly decreases and stabilizes at 1.5 by the fifth epoch, showing a robust link between training and validation performance with low overfitting. In training and validation, accuracy consistently increases, reaching 90% by the 10th epoch. However, the validation accuracy ranges from 25% to 35%, indicating good performance on training data.

**Figure 9:**
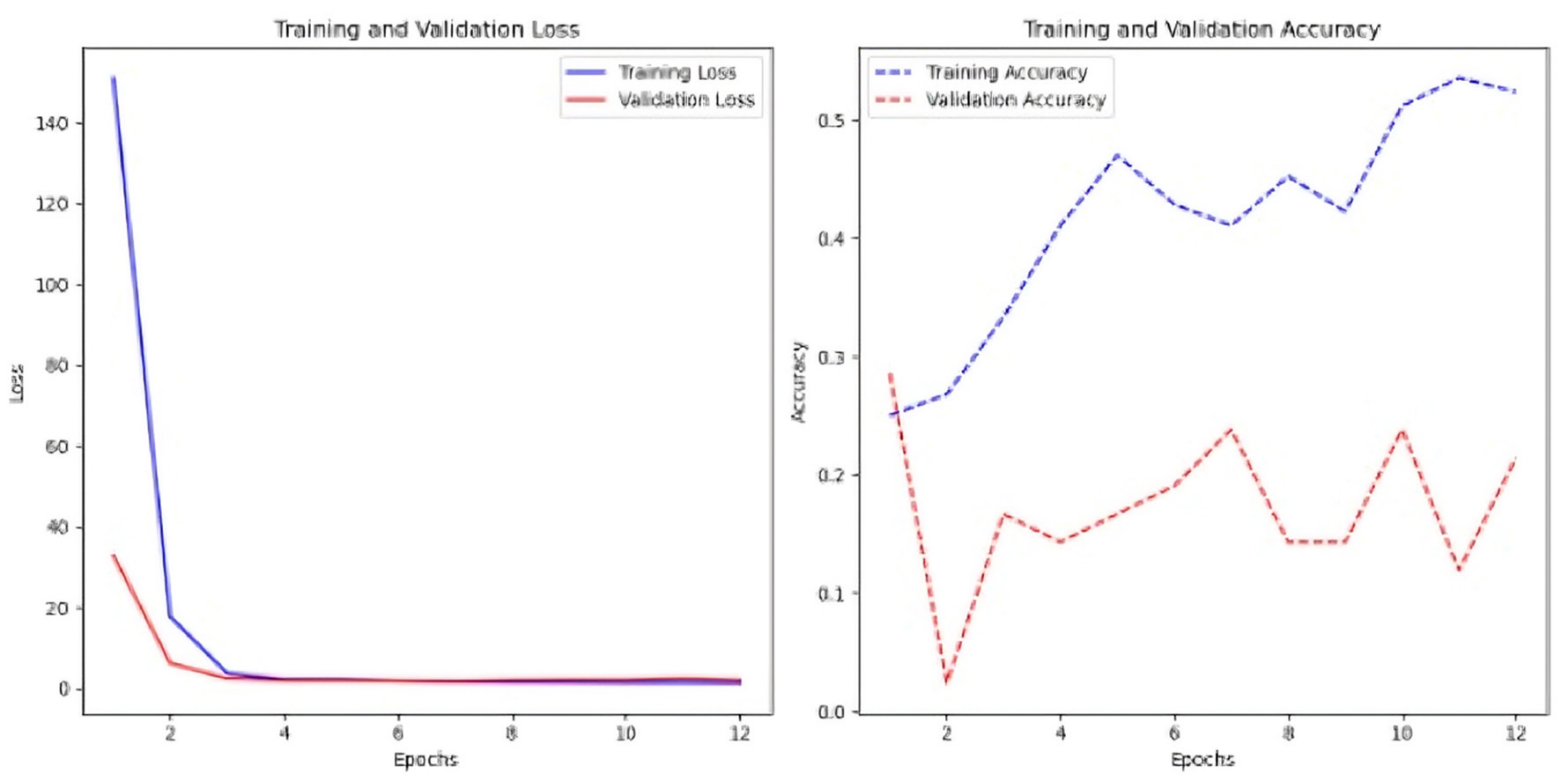
Sc 1 loss an accuracy values in CNN with Keras auto-tuner and SMOTE.

The CNN model was also evaluated using SMOTE (Rachmatullah, 2022) (Figure 9), to address class imbalance (Rachmatullah, 2022), but this approach yielded significantly worse results. The best performance, obtained after tuning with keras auto-tuner using 1500 time-steps per parameter, resulted in an accuracy of 0.214 and a loss of 3.5.

**Figure 10:**
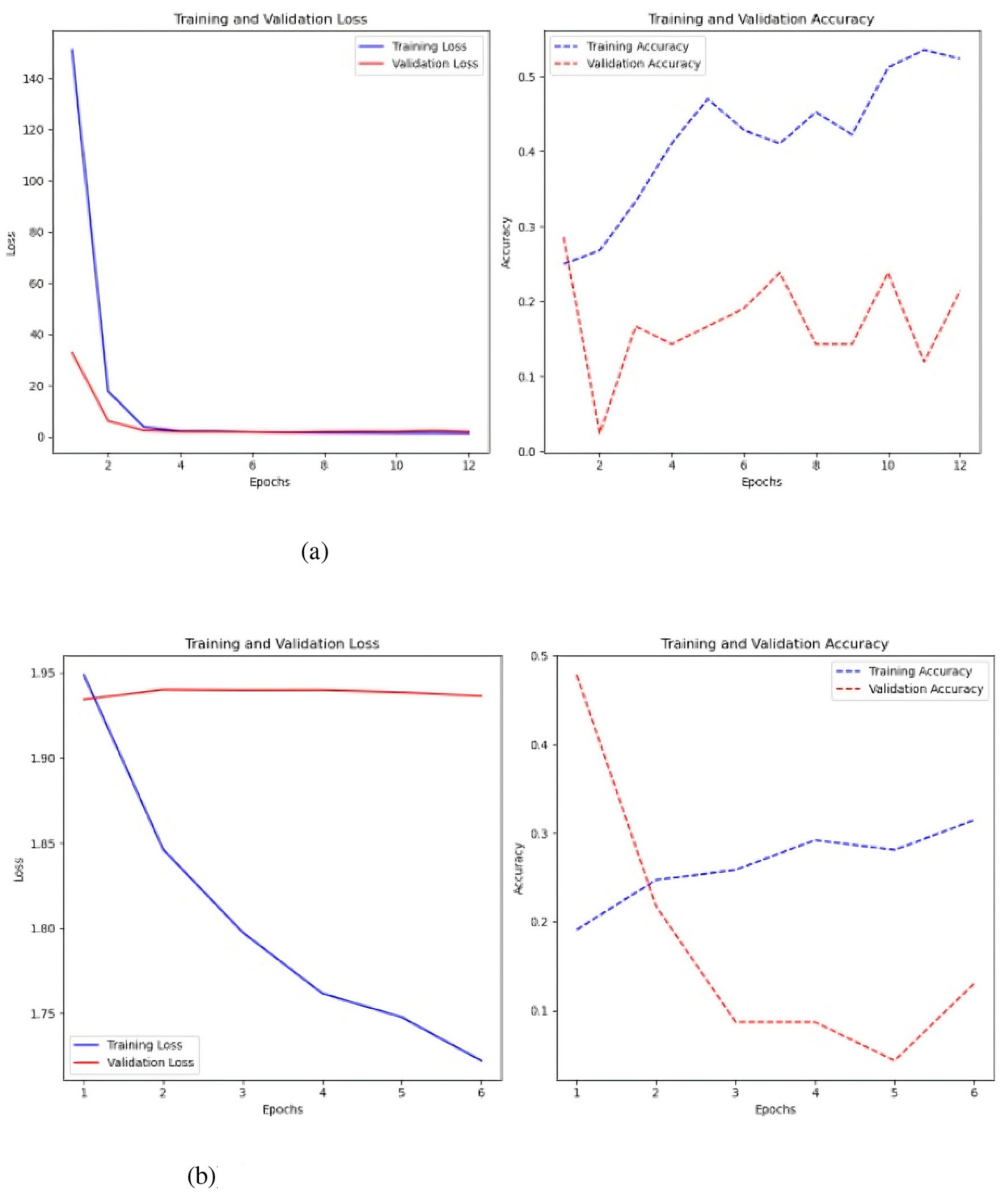
Results of the FCN model with and without the Keras auto-tuner. **(a)** Sc 1 loss an accuracy values in FCN. **(b)** Sc 1 loss an accuracy values in FCN with Keras auto-tuner.

The final model tested was the FCN both with and without the Keras auto-tuner (Figure 10).

Simple FCN performed best at 1500 time steps per time series with 0.35 accuracy and 1.94 loss. The Keras auto-tuner FCN performed best with 500 time steps per parameter, 0.28 accuracy and 2.09 loss. Training loss for basic FCN steadily lowers from 1.95 to 1.7 by the sixth epoch (see to Figure 10a). Improvement and stability at 1.9 in validation loss. This shows the model has trouble predicting new data. A slight drop to 0.45 increases training precision. Once validation accuracy drops to 0.15, it stays between 0.10 and 0.15 for subsequent epochs.

The considerable gap between training and validation accuracy and the marginally lower validation loss suggest overfitting, in which the model performs better on training data than on validation data. FCN with Keras auto-tuner (Figure 10b) shows a continuous decrease in training loss and an increase in validation loss after a given number of epochs. The model may overfit the training data by memorizing the examples rather than generalizing it to new data.

However, attempts to rectify the FCN model's class imbalance by utilizing SMOTE were unsuccessful. After adjusting each parameter with 1500 time steps in the Keras auto-tuner, the optimal performance yielded a loss of 2.13 and an accuracy of 0.25. Among the three models tested, CNN proved to be the most effective for this preliminary strategy.

### 3.2 Modeling—Scenario 2

**Figure 11:**
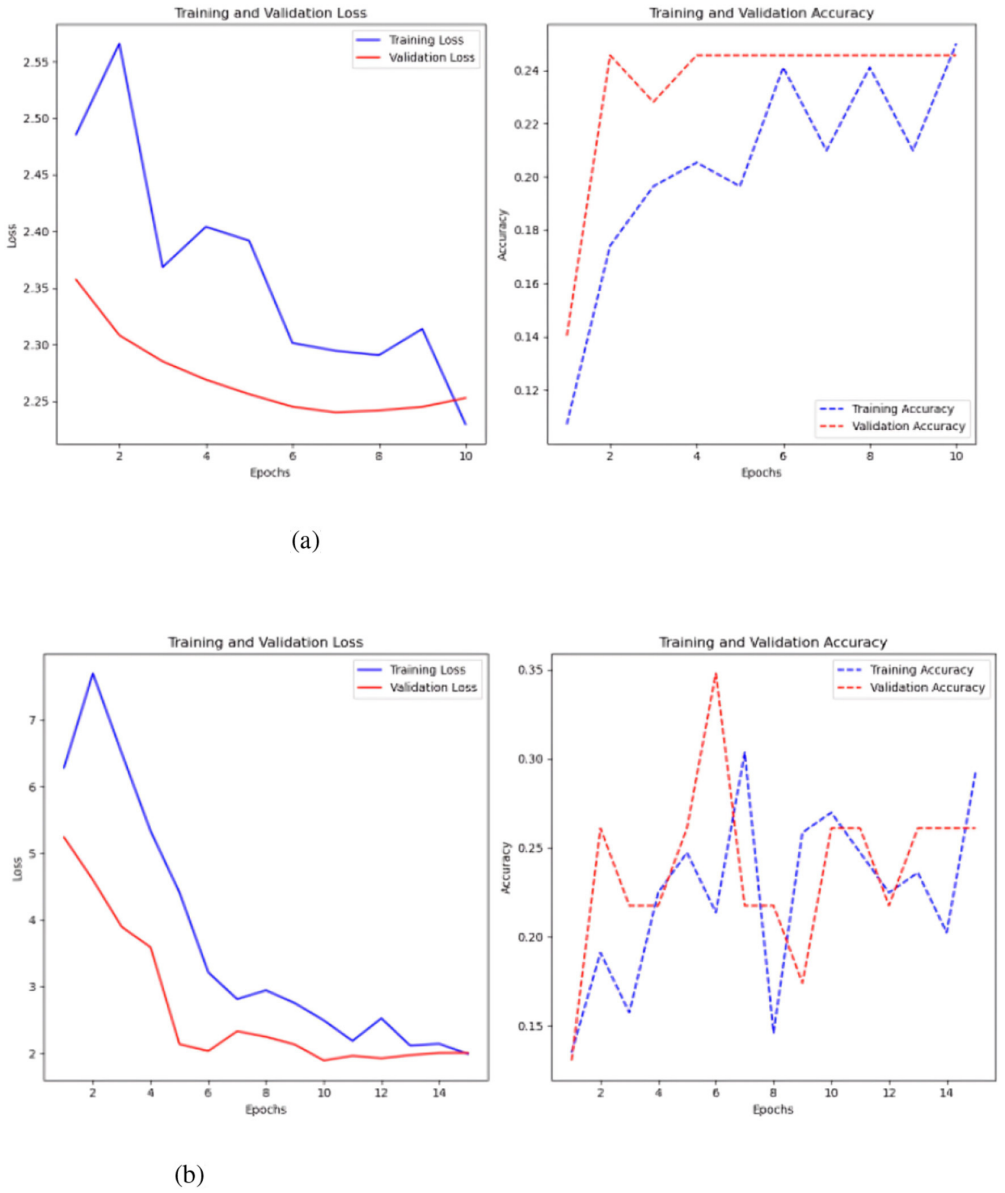
MLP test results, with and without the Keras auto-tuner. **(a)** Sc 2 loss an accuracy values in MLP. **(b)** Sc 2 loss an accuracy values in MLP with Keras auto-tuner.

The second scenario uses ten time-series variables to classify failure. The current time series data is organized in a three-dimensional array with the number of samples, variables, and time steps for each variable, unlike the previous example. This data format keeps the 3-dimensional array size constant across all samples for all time series. The initial model tested was the MLP, both with and without the Keras auto-tuner (see Figure 11).

A 500-step time-series test sans Keras auto-tuner yielded the best results, with an accuracy of 0.23 and a loss of 2.20 (Figure 11b). Keras auto-tuner fared best with 1500 time steps per series, 0.27 accuracy, and 2.12 loss. Different MLP models operate. Untuned models reduce training and validation loss by learning slowly. It is 0.24 accurate. Strong generalization and no overfitting are indicated by a somewhat greater validation accuracy than training accuracy. Keras auto-tuner (Figure 11b) causes increased volatility. Although peaking around 0.3, training and validation accuracy swings, suggesting instability. Accuracy swings indicate overfitting or training sensitivity, but loss drops dramatically.

**Figure 12:**
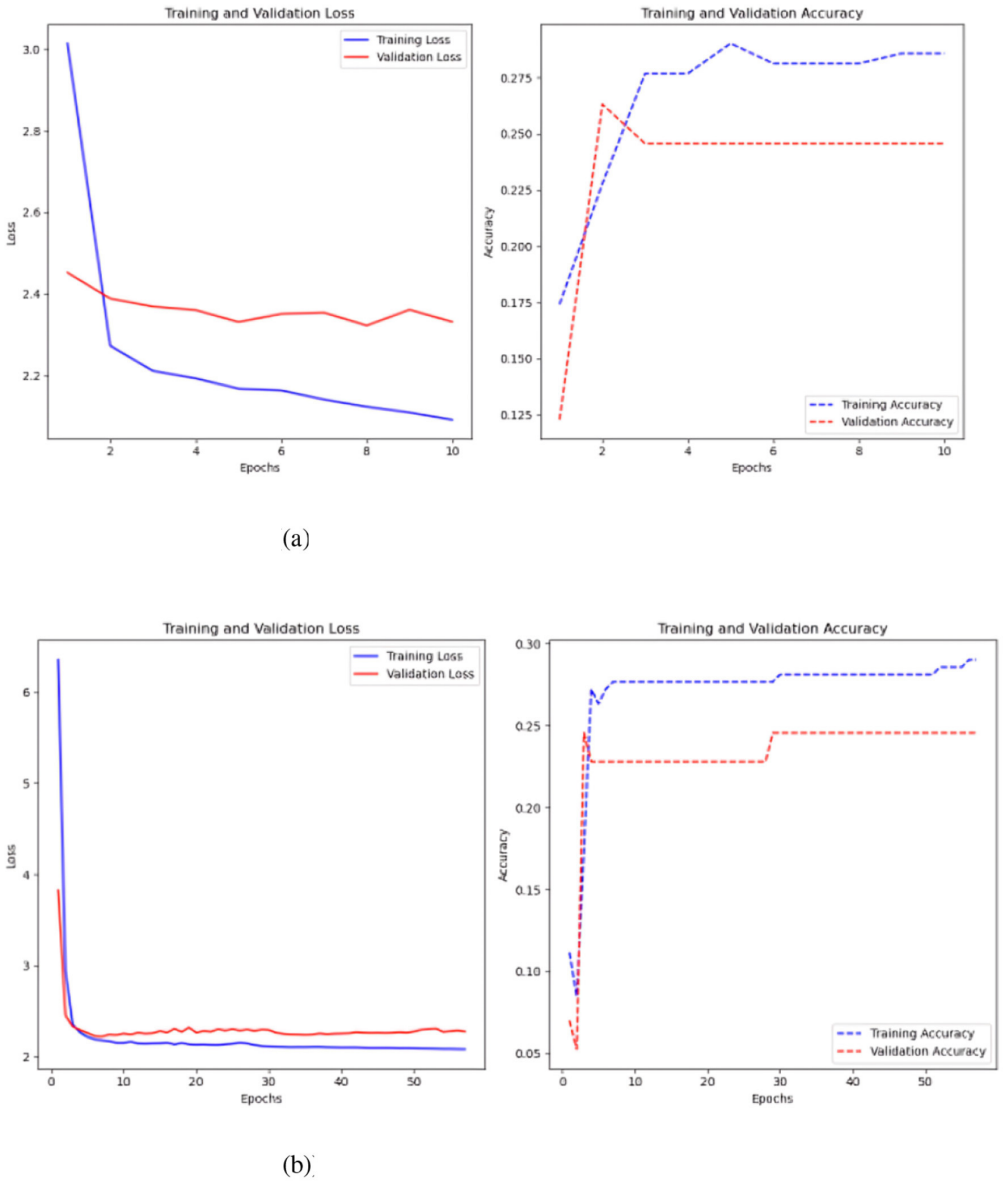
Results of the CNN model test. **(a)** Sc 2 loss an accuracy values in CNN. **(b)** Sc 2 loss an accuracy values in CNN.

The second model tested in this scenario was a CNN, where the architecture from the previous test was retained, with the only modification being a different input data format. Like the previous experiment, the model was evaluated with and without Keras auto-tuner (Figure 12).

Without keras auto-tuner (Figure 12b), 500 time-steps per time series yielded the best accuracy of 0.26 and a loss of 2.32. Using Keras auto-tuner (Figure 12a), the optimal result was achieved with 500 time-steps per time series, but with better accuracy of 0.28 and reduced loss of 2.18. The CNN model without the Keras auto-tuner performs well on the training set but overfits. Poor generalization is shown by a validation accuracy of 0.18 and a training accuracy of 0.27. In comparison, the Keras auto-tuner MLP model generalizes better. The training accuracy peaks at 0.29, and the validation accuracy follows at 0.25, showing higher consistency across datasets.

**Figure 13:**
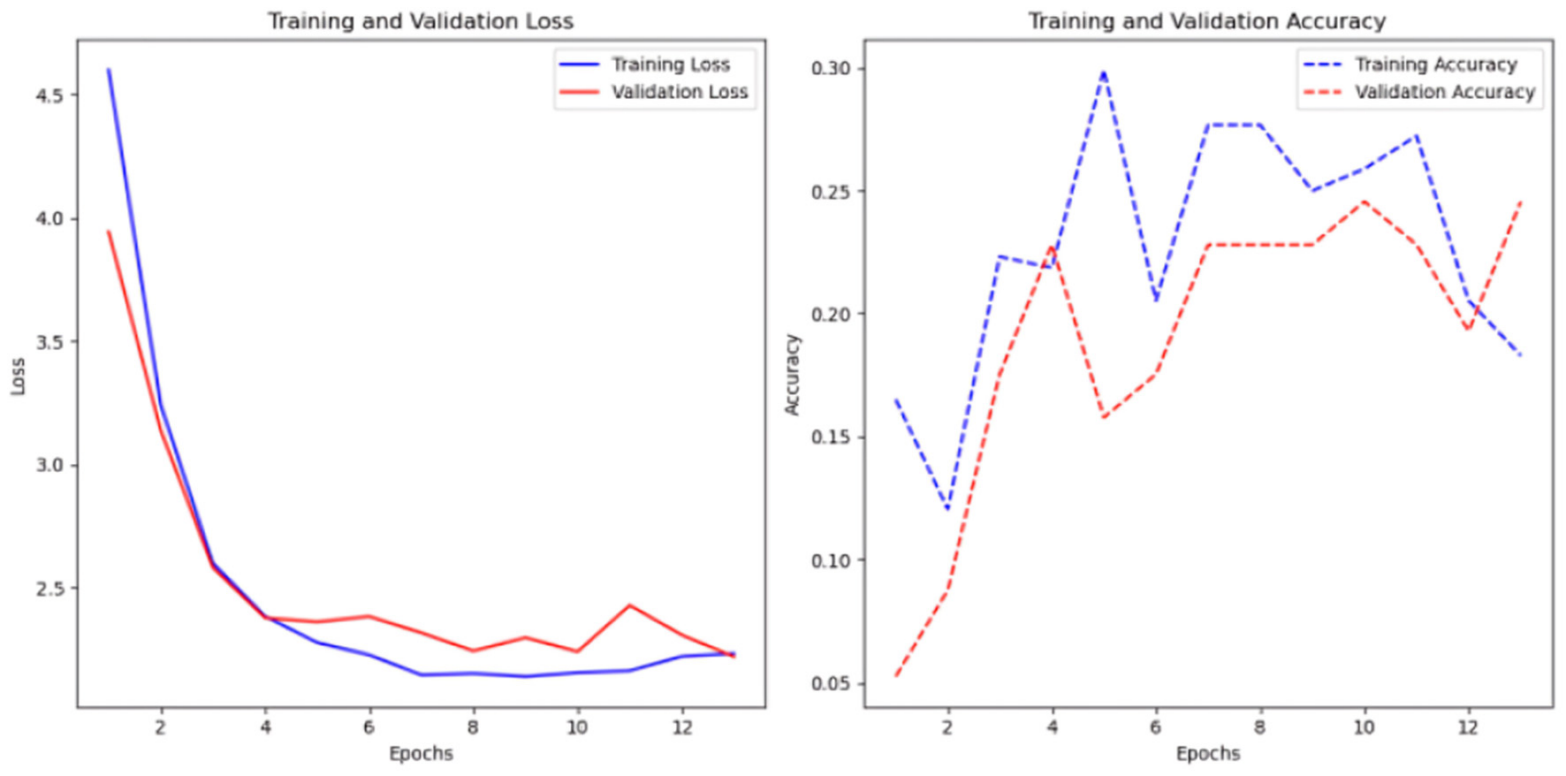
Sc 2 loss an accuracy values in multi-head CNN with Keras auto-tuner.

The model was improved and evaluated via Keras AutoTuner, a hyperparameter tuning library. Optimal performance was attained after thorough tuning with 500 time steps for each time series variable. This combination had an accuracy of 0.31 and a loss value of 2.23. Figure 13 demonstrates a distinct downward trend, with training and validation loss consistently declining as the model trains, signifying that the model is acquiring knowledge and enhancing its fit to the data.

Model convergence occurs during the 10th epoch when training and validation loss levels stabilize and decrease. A close final loss value means the model did not overfit the training data. Training loss is slightly lower than validation. However, the accuracy plot, notably the validation accuracy, fluctuates wildly. Initial training and validation accuracy rises but loses stability. The model's accuracy improves steadily but remains around 30% post-training, indicating little predictive capability despite capturing data trends. The validation accuracy shows even greater peaks and drops, proving the model cannot generalize to new data.

**Figure 14:**
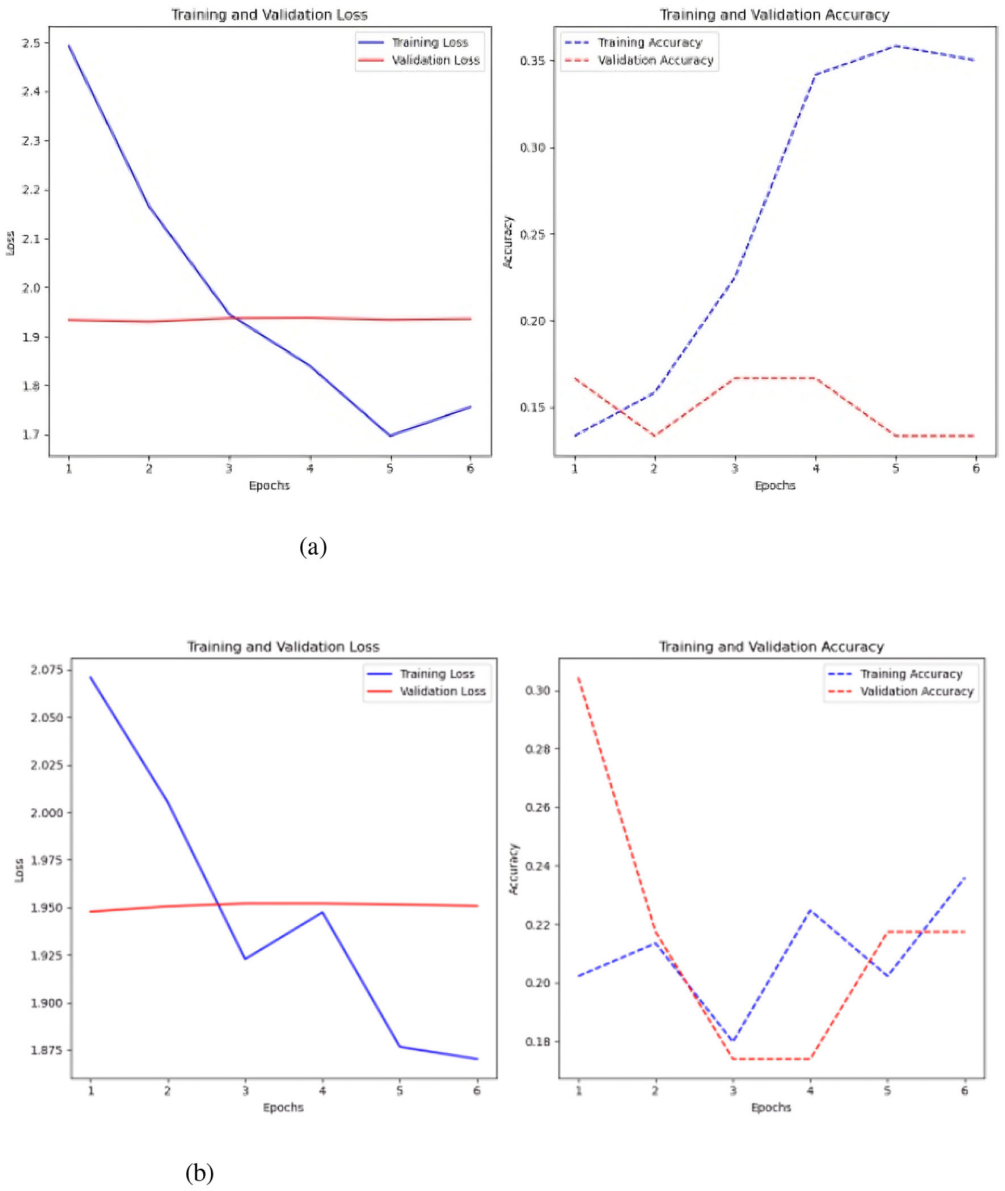
Results of the FCN model with and without the Keras auto-tuner. **(a)** Sc 2 loss an accuracy values in FCN. **(b)** Sc 2 loss an accuracy values in FCN with Keras auto-tuner.

The FCN was the last model tried in this scenario; it used the identical architecture as the previous experiment, with the input data format being the sole difference. Like the earlier case, the model was assessed with and without the Keras auto-tuner (Figure 14).

Without Keras auto-tuner, 1,000 time steps per time series resulted in the best performance, with an accuracy of 0.31 and a loss of 1.85 (see Figure 14b), Keras' auto-tuner chose the ideal setup with 1,500 time steps per time series, obtaining 0.28 accuracy and 1.96 loss. The Keras auto-tuner examined numerous configurations. However, the optimal results without tuning had slightly higher accuracy and lower loss.

Early epochs without Keras auto-tuner lose less training. Model convergence occurs when the loss curve flattens in the sixth epoch. However, validation loss remains around 1.9 during training, indicating poor validation set generalization. The rising training-validation loss gap suggests overfitting. The accuracy plot shows that training accuracy approaches 35%, whereas validation accuracy remains at 12–15%. In the second example, Keras auto-tuner reduces model training loss progressively (Figure 3). The previous model validation loss was 1.9.

Though narrower, the training-validation loss gap shows less overfitting than the untuned model. Training accuracy is 15–25%. However, validation accuracy fluctuates mid-training and recovers. Despite oscillations, validation accuracy is 15–25%, comparable to the untuned model. Training data improves both models. However, validation set performance is uneven and overfitting.

In this scenario, among the four models tested, the multi-head CNN stands out in its ability to learn from multivariate time-series data, particularly for the classification task.

## 4 Discussion

This section presents the discussion of the results, comparing the different scenarios. This comparison is made using Table 1, which was created to display the best results obtained from each model tested across all scenarios. This table presents the top-performing result for each model under each scenario, along with the corresponding number of time-steps for the 10 variables in time-series format. The columns labeled “Size of TS-1” and “Size of TS-2” represent the length of the time-series data for scenario 1 and scenario 2, respectively.

**Table 1:** Best results scenario comparation.

**Models**	**Scenario 1**	**Size of TS- 1**	**Scenario 2**	**Size of TS-2**
MLP	0.32	500	0.27	1,500
CNN	0.34	500	0.28	500
Multi-head CNN	Not applicable		0.30	500
FCN	0.36	1,500	0.31	1,000

Table 1 indicates that all models exhibited comparable performance in both cases, with negligible outcome changes. Notably, the models' performance at 500 time steps is significant, especially considering the heightened difficulty of this configuration. The assignment required guessing 11 distinct classes, resulting in a baseline accuracy of around 0.09 (1/11) for a random pick.

Despite comparable performance, the models' 500-time-step results stand out, where the classification problem is more complex due to the increasing number of classes. These models learned from time series data and predicted several classes with better-than-random accuracy even in difficult categorization situations.

Low results were primarily due to poor data quality for the models. The dataset has few samples and 10 time-series variables with low predictive ability, making it difficult for models to identify target groups. Better feature selection and engineering can extract more relevant and informative features from time-series data.

The development of this work enables us to present several contributions that involve the successful integration of sensory data from time series with historical FMEA records, which categorize equipment failures and previous corrective actions, representing a significant achievement of this research.

The successful integration of time-series sensory data with historical FMEA records, which categorize past equipment failures and corrective actions, represents a key achievement of this research. Data preprocessing techniques were employed to clean, standardize, and transform the sensor data, ensuring consistency and readability for the models. Additionally, various methods were applied to format the time-series data to allow models to interpret the records in multiple ways, thus facilitating a thorough analysis of each model's predictive capabilities.

It is also important to note that the categorization of repair actions required careful refinement, including the consolidation of similar actions to reduce the number of classes. This step was essential to improve the models' ability to generalize different failure modes while providing accurate and actionable recommendations. By structuring the data in this way, the research was able to effectively test and compare various approaches to multi-class classification, further contributing to a better understanding of the relationship between sensory data and equipment failures.

Finally, this work provided valuable insights into the practical application of AI in real-world scenarios. The challenges encountered during model development revealed important lessons about the complexities of working with AI in this industrial context, pointing to areas for future research and improvement. Despite the setbacks, the project was an essential learning experience in applying artificial intelligence to operational processes.

The challenges encountered during model development revealed key lessons about the intricacies of working with AI in this industrial context, pointing to areas for future research and improvement. Despite the setbacks, the project was an essential learning experience in applying artificial intelligence to operational processes.

## 5 Conclusion

This paper investigated whether machine learning models may help maintenance teams predict production line equipment breakdown repair processes. Ten industrial equipment time series variables and 1441 historical corrective failures were examined. This study used time-series sensory data and FMEA records to categorize equipment failures and corrective actions. Filtering, standardizing, and translating sensor data ensured model clarity. Time series data came in many ways, so models interpreted them differently.

Repair activity classification requires purposeful improvement, combining related acts to minimize classifications. The models generalized across failure scenarios and produced actionable recommendations throughout this step. This data format compares multi-class classification methods to improve sensory data and equipment failure knowledge. Models surpassed random chance under challenging cases with multiple failure types. Though the dataset is flawed, the strategy sounds promising. MLP, CNN, Multi-head CNN, and FCN found intriguing time-series patterns in various scenarios.

The results indicate that, among the three models evaluated, the CNN achieved the best performance for this initial approach. However, it is evident that the models struggled with generalization, as their ability to predict unseen data was limited. This issue is likely due to the relatively small number of time series, which may not provide sufficient variability for the models to learn effectively. Additionally, combining multiple time series into one dataset could have introduced complexity, making it more difficult for the models to differentiate between distinct parameters or temporal patterns.

As a result, this approach may have hindered the model's ability to capture unique trends and relationships within each individual time series, impacting their overall predictive accuracy. In this scenario, among the four models tested, the multi-head CNN stands out in terms of its ability to learn from multivariate time-series data, particularly for the classification task. While the accuracy results in the test set were not significantly better than the other models, the training graphs indicate that the multi-head CNN exhibits stronger learning capabilities.

This suggests that the model can capture more complex patterns during training, even though this advantage did not fully translate to better performance in the test results. The challenge with achieving higher test accuracy lies in the nature of the data itself. The time-series variables used appear to have limited correlation with the target classes or fault types, making it difficult for the model to learn meaningful relationships. Additionally, the dataset suffers from a lack of sufficient occurrences of corrective faults, leading to an imbalance in the data. This scarcity of fault-related samples limits the model's ability to generalize and accurately predict these rare events.

This issue of insufficient data, especially about corrective faults, negatively impacted the performance of all four models tested. The limited sample size and the inherent complexity of the time-series variables reduced the models' ability to effectively differentiate between the classes. This problem mirrors the challenges observed in the previous scenario, where the models similarly struggled to perform well due to the lack of robust and diverse training examples. Addressing this issue would likely require either the collection of more comprehensive fault data or the use of advanced techniques, such as data augmentation or synthetic data generation, to improve the models' ability to generalize.

Low performance shows that data preprocessing and model development must be enhanced for more accurate predictions. The complexity of predicting corrective failures, particularly with limited and weakly predictive data, suggests that incorporating additional data sources or employing more sophisticated techniques will be crucial for significantly improving prediction accuracy.

Future work could improve input data quality through feature engineering and selection, and add data sources to improve the model's forecasting powers. Synthetic data synthesis may be considered to handle limited data. Transformers, ensemble learning, and hyperparameter fine-tuning could increase performance. Transfer learning and attention methods may help the model capture time-series data's temporal dependencies.

Current models can forecast corrective failures, but their accuracy is limited. Maintenance teams need broader approaches to suit their needs. Enhancing and expanding the existing models can lead to a more accurate and dependable system that supports maintenance operations and reduces downtime.

## Data Availability

The datasets presented in this study can be found in online repositories. The names of the repository/repositories and accession number(s) can be found in the article/supplementary material.
